# Accelerated abdominal lipid depletion from pesticide treatment alters honey bee pollen foraging strategy, but not onset, in worker honey bees

**DOI:** 10.1242/jeb.245404

**Published:** 2023-04-12

**Authors:** Megan Elizabeth Deeter, Lucy A. Snyder, Charlotte Meador, Vanessa Corby-Harris

**Affiliations:** ^1^ Department of Entomology and Insect Science, University of Arizona, Tucson, AZ 85721-0036, USA; ^2^ Carl Hayden Bee Research Center, USDA-ARS, Tucson, AZ 85719, USA

**Keywords:** *Apis mellifera*, Adipose tissue, Fat body, Foraging, Pesticides

## Abstract

Honey bee abdominal lipids decline with age, a change thought to be associated with the onset of foraging behavior. Stressors, such as pesticides, may accelerate this decline by mobilizing internal lipid to facilitate the stress response. Whether bees with stressor-induced accelerated lipid loss vary from controls in both the onset of foraging and nutritional quality of collected pollen is not fully understood. We asked whether stressors affect foraging behavior through the depletion of abdominal lipid, and whether stress-induced lipid depletion causes bees to forage earlier and for fattier pollen. We tested this by treating newly emerged bees with one of two pesticides, pyriproxyfen (a juvenile hormone analog) and spirodiclofen (a fatty acid synthesis disruptor), that may affect energy homeostasis in non-target insects. Bees fed these pesticides were returned to hives to observe the onset of foraging behavior. We also sampled foraging bees to assay both abdominal lipids and dietary lipid content of their corbicular pollen. Initially, spirodiclofen-treated bees had significantly more abdominal lipids, but these declined faster compared with controls. These bees also collected less, yet more lipid-rich, pollen. Our results suggest that bees with accelerated lipid decline rely on dietary lipid content and must collect fattier pollen to compensate. Pyriproxyfen treatment reduced the age at first forage but did not affect abdominal or collected pollen lipid levels, suggesting that accelerated fat body depletion is not a prerequisite for precocious foraging.

## Teaser

**Highlighted Article:** Honey bees exhibit a gradual decline in abdominal lipid that can be accelerated by stress; this pesticide-induced accelerated lipid decline shifts foraging preference to fattier pollen.

## INTRODUCTION

Obesity is a growing public health crisis that threatens the longevity and well-being of those affected. Poor diet, lack of exercise and chronic stress are the most significant factors contributing to excess lipid accumulation (Blüher, 2019). Although obesity can be treated with exercise and a balanced diet, many patients report tremendous difficulty in losing weight and maintaining weight loss, particularly those with higher BMIs (Carnell and Wardle, 2008). Individuals with higher proportions of body fat, or adipose tissue, oftentimes experience lower levels of satiety, despite typically consuming a diet of caloric excess (Blüher, 2019; Boutelle et al., 2020). This counterintuitive behavioral response may make animals more vulnerable to obesity (McMillen et al., 2005). Understanding the physiological component behind these seemingly maladaptive feeding behaviors that further the obese state is key to combating this public health crisis.

From an evolutionary perspective, the ability to store excess dietary energy in adipose tissue can be seen as a selective advantage (McMillen et al., 2005). Most organisms have a homeostatic set point that favors lipid storage, so that the energy stored as fat and glycogen can be mobilized during times of nutritional scarcity (McMillen et al., 2005; Arrese and Soulages, 2010; Nelson et al., 2021; Charmandari et al., 2005). External stressors can disrupt homeostasis, triggering a stress response that acts to restore optimal internal conditions (Chovatiya and Medzhitov, 2014). One example of a behavioral response to stress is the increased consumption of palatable, calorically dense foods (Boutelle et al., 2020; Ortolani et al., 2011). While increased consumption can be beneficial by providing additional energy for the stress response, in excess it can lead to inflammation and other metabolic diseases, such as obesity or insulin resistance (Kershaw and Flier, 2004). Such pathologies have become commonplace in modern human society, as people in industrialized nations experience less nutritional scarcity, an increased availability of heavily processed and highly palatable foods, and an increase in socioeconomic stress (Blüher, 2019; Capehart and Wisman, 2013).

While subject to conscious control, food-intake behavior is influenced by a variety of physiological signals that can further affect dietary decisions, sometimes at a cost to the individual. Adipose tissue can be a source of such signals and is emerging as an endocrine tissue in its own right. To understand how body fat influences food-intake behavior, it is helpful to first consider the evolutionary and physiological role of adipose tissue. Adipose tissue not only serves as an energetic reservoir but also operates in tandem with other effector organs to relay messages concerning homeostasis (Kershaw and Flier, 2004; Agustí et al., 2018). Adipose tissue synthesizes and secretes appetite-related hormones that relay messages regarding satiety, such as leptin (in vertebrates) and adipokinetic hormone (in invertebrates) (Arrese and Soulages, 2010; Nelson et al., 2021; Kershaw and Flier, 2004; Baile et al., 2000). These signals are received by the central nervous system (CNS) to mediate a physiological and behavioral response to restore homeostasis (Charmandari et al., 2005). Chronic stress can induce lasting changes to appetitive regulation irrespective of an organism's actual energy expenditure (Agustí et al., 2018; Xiao et al., 2020). We are just beginning to understand the physiological mechanisms connecting stress to changes in food-intake behavior. This topic is particularly challenging to study because the links between adipose tissue and behavior are complex and difficult to test and experimentally manipulate within human subjects.

Insect models are valuable tools for elucidating the connection between adipose tissue and behavior. Insects have short generation times compared with vertebrates. Additionally, their nutritive status is far easier to manipulate in laboratory settings, as they can be reared on chemically controlled diets with relative ease. The majority of insect lipid is stored within the fat body, an amorphous collection of adipose tissue found in both the abdomen and head, although the majority of the fat body exists in the abdomen (Arrese and Soulages, 2010). In addition to fat storage, the insect fat body is also a dynamic metabolic organ that is highly receptive to endocrine signaling. In *Drosophila*, increased expression of the stress hormone octopamine substantially decreases the fat body, in tandem to decreasing food intake, appetite and metabolic activity (Li et al., 2016).

Honey bees (*Apis mellifera*), in particular, are an excellent system to understand this stress–fat behavior axis. Honey bee workers exhibit temporal polyethism, where their role within the colony changes with age (Wilson and Hölldobler, 2005). Workers begin their adult life in the hive, exhibiting brood- and queen-care (‘nurse’) behaviors within ∼2–3 weeks post-emergence. As they age beyond 3 weeks old, they leave the hive (‘forager’) in search of water, nectar and pollen for the colony (Winston, 1987). During this transition, workers experience a decline in fat body lipid and an increase in juvenile hormone levels (Toth et al., 2005; Toth and Robinson, 2005; Sullivan et al., 2000). Juvenile hormone negatively affects expression of vitellogenin, an egg yolk precursor protein that promotes stress resilience, longevity and reproduction (Corona et al., 2007). Therefore, higher juvenile hormone titers are linked to a reduction in stress resilience and accelerated aging, possibly via depletion of lipid reserves. Honey bees are not the only organism where a decline in internal lipid precedes the transition into foraging behavior. Other Hymenoptera species also exhibit a similar link between fat body and foraging onset, such as the eusocial clonal ant (*Platythyrea punctata*) and the primitively eusocial paper wasp (*Polistes metricus*) (Daugherty et al., 2011; Bernadou et al., 2020).

Although the nurse-to-forager transition is socially regulated, external stressors can prompt workers to forage earlier, a phenomenon known as precocious foraging (Robinson, 1987; Schulz et al., 1998; Toth et al., 2005; Goblirsch et al., 2013). While recruitment of precocious foragers immediately increases the food intake for a colony by increasing the foraging workforce, it is associated with higher worker mortality, which can further accelerate colony failure (Perry et al., 2015; Woyciechowski and Moroń, 2009). The accelerated development of precocious foragers may also come at a cost to cognition. Precocious foragers display lapses in spatial memory and fine-scale searching behavior (Ushitani et al., 2016), both processes crucial to foraging success. It is also possible that these foragers utilize internal nutritional stores at a faster rate and, therefore, have metabolic limitations on the duration of foraging trips.

Previous work indicates that stressed bees forage earlier (Perry et al., 2015; Woyciechowski and Moroń, 2009; Khoury et al., 2011) and experimentally induced fat body depletion is associated with precocious foraging (Toth et al., 2005), so stress-induced fat body depletion may be the main causative factor linking stress to changes in foraging behavior. Within the eusocial insects, fat body lipid decline has largely been studied within the context of foraging onset, yet rarely within the scope of changes to foraging strategy. Whether stressed foragers also shift their foraging strategy towards collecting more nutrient-rich pollen to compensate for abdominal lipid depletion remains unclear. Stressed bees possibly have a lower energetic capacity, as a result of their mobilization of reserves to fuel the stress response (Arrese and Soulages, 2010), making them more susceptible to the physical demands of foraging, and can complete fewer foraging trips (Sullivan et al., 2000). We speculate that they must maximize their foraging yield by collecting pollen with a higher lipid content, as it has a higher caloric value per gram than protein.

We examined these questions using one of many stressors that honey bees are likely to encounter in the field – insect growth regulator pesticides (IGRs) (Johnson et al., 2010). IGRs interfere with the development and reproduction of target crop insect pests. They are generally regarded as pollinator-safe because of their specificity (Desneux et al., 2007) but may negatively affect hormone signaling and nutrient storage in non-target species (Meola et al., 2000; Rivero et al., 2011). Two IGRs, pyriproxyfen and spirodiclofen, are agriculturally relevant and may affect energetic homeostasis (Johnson et al., 2010). Pyriproxyfen is a juvenile hormone mimic that may accelerate the nurse-to-forager transition, a change that is associated with lipid loss. Spirodiclofen is a fatty acid synthesis disruptor and may also accelerate this transition by directly inhibiting lipid synthesis. Here, we focused on the abdominal fat body as a proxy for nutritional lipid status. We asked whether these IGR stressors induced abdominal lipid loss and altered worker foraging behavior. If stress increases the likelihood of precocious foraging (Toth et al., 2005; Perry et al., 2015; Woyciechowski and Moroń, 2009; Khoury et al., 2011), and if precocious foragers have been shown to be cognitively deficient (Ushitani et al., 2016), in that they exhibit clear deficits in spatial memory associated with foraging success, then we expect stressed foragers to collect less pollen (smaller corbicular pellets) per foraging trip. As we expected these stressors to act directly on the fat body, we also expected foragers to collect more, fattier pollen to compensate for stress-induced abdominal lipid reduction.

## MATERIALS AND METHODS

### Source bees

All experiments were conducted at the Carl Hayden Bee Research Center in Tucson, AZ, USA. Colonies were headed by *Apis mellifera ligustica* Spinola 1806 queens from a commercial breeder in California. Field experiments (experiments 3 and 4) used queen-right colonies that had an even brood distribution and no visible signs of disease or stress. Capped brood frames from these colonies were removed and placed in a temperature- and humidity-controlled incubator (33±1°C, 50% humidity) overnight with no light. Adults that emerged within an 18 h period were gently brushed from these frames prior to treatment.

### Pesticide treatment

Newly emerged bees were randomly assigned to one of the three treatment groups: pyriproxyfen, spirodiclofen or a solvent control. Pesticides (PESTANAL^®^ analytical standard, Sigma-Aldrich, St Louis, MO, USA) were dissolved in acetone and then suspended in sugar water. Each bee was fed 2 μl of the treatment–sugar water mixture by hand with a micropipette. For all experiments, the bees were fed once at adult emergence, reflecting an acute exposure from contaminated nectar shortly after emergence (Johnson et al., 2010; Fries et al., 2013). We did not test the effects of chronic exposure. Feeding bees once allowed for tight control of the individual pesticide dose, which was important because we observed individual behavior and lipid levels. For the survival analysis experiment (experiment 1), bees were fed a high (42 mg l^−1^), medium (21 mg l^−1^) or low (10.5 mg l^−1^) dose of pesticides, with the final amount of pesticide being fed to each bee totaling 84 ng (high), 42 ng (medium) or 21 ng (low). For experiments 2 and 3, pesticide-treatment groups were fed only the high (42 mg l^−1^) concentration of both spirodiclofen and pyriproxyfen.

### Experiment 1: do IGRs increase mortality in caged bees?

We first examined the survivorship of bees fed one of the two IGR pesticides at three field-relevant doses or a negative acetone control (Johnson et al., 2010). To maximize the number of treated bees surviving long enough to reach foraging age in experiments 2 and 3, we aimed for a treatment concentration where 50% or more bees survived to 14 days old, the average age that bees initiate foraging (Winston, 1987). Newly emerged worker bees (≤18 h old) were brushed from frames and placed individually into empty 1.5 ml tubes for 1 h prior to feeding. Bees were then removed from the tubes and fed 2 μl by pipette of either a high (42 mg l^−1^), medium (21 mg l^−1^) or low (10.5 mg l^−1^) dose of pesticide dissolved in acetone and mixed with sugar water, or a negative control containing only acetone and sugar water. After feeding, bees were again isolated in 1.5 ml tubes for another hour to prevent them from exchanging treatment through trophallaxis with each other (Fries et al., 2013). Bees that successfully consumed the entire 2 μl of treatment without regurgitating were placed into bioassay cages (11.5 cm×7.5 cm×16.5 cm) with 50 other bees of the same treatment and dose. Bees were treated once to tightly control the pesticide dose (see above). Bees in the cages were provided with distilled water, 30% (w/v) sucrose water, and pollen patty *ad libitum*, consisting of a 1:1:1 ratio of bee-collected pollen, table sugar and drivert sugar (8% sucrose, fructose+92% sucrose). Pesticide-free bee-collected pollen was purchased in bulk (Great Lakes Bee Supply, Galesburg, MI, USA). Dead bees were removed and mortality was recorded daily for a minimum of 3 weeks or until survivorship fell below 10%.

### Experiment 2: do IGRs affect the age at first forage?

Field experiments were conducted in August 2019 (trial 1, 2 hive replicates) and May 2020 (trial 2, 3 hive replicates). Each replicate hive contained 100 bees per treatment (pyriproxyfen, spirodiclofen or a solvent control), placed into the same hive. The bees were fed the high dose (42 mg l^−1^) of either pyriproxyfen or spirodiclofen, or the solvent control, as in experiment 1. Bees that successfully consumed the entire treatment were labeled with color-coded (by treatment) and numbered tags in order to track individuals from each treatment group. After the bees were treated and tagged, they were placed into the experimental colony. Hive entrances were extended using a Styrofoam landing platform with a Plexiglas cover to better observe and record the ID numbers of foragers. Observations were conducted at the hive entrances every morning (starting at 4 days of age) between 07:00 h and 12:00 h at 15 min intervals.

**Fig. 1.**
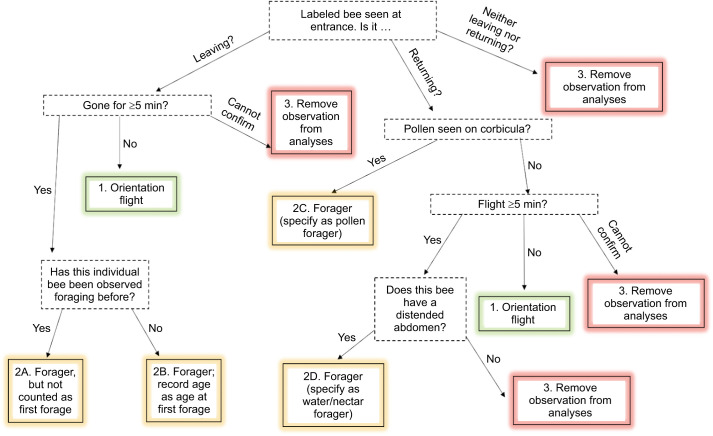
**Classification of foraging trips for experiment 2.** The flow chart depicts the process of classifying foraging trips observed in experiment 2 as (1) orientation flights or (2) foraging trips, including whether a trip was counted as the first recorded age of first forage and whether the forager collected pollen or exclusively water/nectar, and (3) the circumstances in which an observation was removed from the dataset.

We took note of the treatment group and ID number of every bee observed at the hive entrance and recorded (1) whether they were leaving or returning, (2) the duration of their flight (if seen both leaving and returning to the hive entrance) and (3) if returning, whether they had pollen in their corbicula or a distended abdomen, indicative of water and nectar foraging (Chang et al., 2015). Prior to the analyses, observations were delineated into orientation flights and foraging trips based on our criteria (Fig. 1). Orientation flights are shorter, pre-foraging trips when individuals prepare for the navigational demands of longer foraging trips (Winston, 1987; Capaldi and Dyer, 1999; Prado et al., 2020). The intention for orientation flights is not necessarily to return with pollen, water, nectar or other materials for the hive but rather for the bee to navigate the environment outside the hive. Foraging trips were separated into two categories: leaving or returning. Individual bees seen leaving the hive for the first time and not returning within 5 min were counted as first-time foragers. Returning bees were further separated into two categories: pollen foragers or water/nectar foragers (see above). If any of these criteria were not confirmed within the 15 min observation period, that individual was removed from the dataset entirely. For example, if a bee was seen leaving the hive 14 min into the 15 min observation period, this observation was discarded, as it could not be confirmed whether it went on an orientation flight or a foraging trip, depending on the duration of the flight.

### Experiment 3: do IGR-treated pollen foragers have fewer abdominal lipids and collect less pollen?

Experiments examining the lipids present in the abdomens of returning foragers and their collected pollen were conducted in August 2020 (trial 1, 2 hive replicates) and May 2021 (trial 2, 3 hive replicates). For each hive replicate, approximately 250 individuals were fed per treatment type. Bees were fed as per experiments 1 and 2, and painted with a water-based marker (one color per treatment) prior to being placed into the hive. Collections took place every morning within the same time frame as experiment 2. For this experiment, we were focused exclusively on pollen foragers, as dietary lipid comes entirely from collected pollen. We did not collect water and nectar foragers. These returning foragers were collected and flash-frozen in liquid nitrogen and stored at −80°C until they were processed.

For each forager, the corbicular pollen was completely removed from both hindlegs under a microscope, dried and weighed. The abdominal carcasses of these frozen foragers were dissected, leaving only the exoskeleton and adhering tissues that included the fat body. Each individual dissected abdominal carcass was dried and weighed (mg) (Human et al., 2013). Dried pollen and abdominal carcasses were twice subjected to a Folch extraction and assayed for total lipid content by the sulfuric acid–vanillin–phosphoric acid assay (Van Handel, 1985). All reagents were obtained from Sigma-Aldrich. Sample absorbance was measured using a plate reader (BIOTEK Synergy HT™, Agilent, Santa Clara, CA, USA) and absorbance was evaluated against a standard curve of samples containing known amounts of vegetable oil dissolved in chloroform. Samples below the limit of detection were discarded prior to the analyses.

### Statistical analyses

All statistical analyses were performed using R (https://cran.r-project.org). Graphs were generated using the following R packages: ggplot2 (https://cran.r-project.org/package=ggplot2), cowplot (https://cran.r-project.org/package=cowplot) and ggsignif (https://cran.r-project.org/package=ggsignif). Log-rank survival curve differences between treatment levels of each pesticide were computed and plotted using the survival (https://cran.r-project.org/package=survival) and survminer (https://cran.r-project.org/package=survminer) packages. The average age at first forage and orientation flights were analyzed using a generalized linear mixed model (GLMM) with age as the dependent variable, treatment as the fixed predictor variable, hive as a random effect, and a gamma link function. A logistic regression was used to analyze the likelihood of a returning forager carrying pollen or water/nectar, with treatment as the predictor variable and hive as a random effect. Leaving foraging trips were not included in the logistic model, as they could not be confirmed within the 15 min observation period if the bees ended up foraging for pollen or for water/nectar. Lipid data were analyzed using a mixed model analysis of variance (ANOVA) as well as linear models. Lipids were the dependent variable, treatment and age were fixed predictor variables, and hive was a random effect. We tested whether forager abdominal lipids and corbicular pollen lipids were influenced by pesticide treatment and the age of the forager, as well as an interaction between the treatment and forager age. All lipid measurements were log-transformed prior to analyses. Pairwise comparisons were made using Tukey's HSD *post hoc* test using the R package emmeans (https://cran.r-project.org/package=emmeans).

## RESULTS

### Survival

**Fig. 2.**
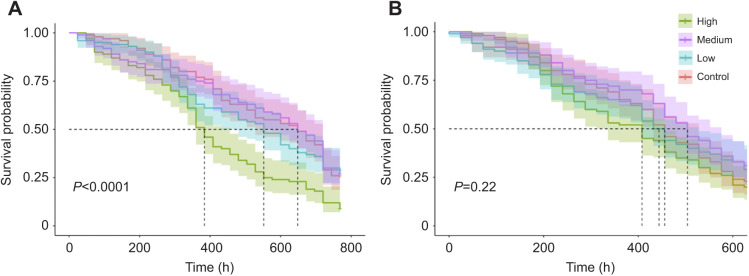
**Survival of caged bees orally exposed to pesticides at adult emergence.** Survival curves indicate the number of caged bees exposed to a high (42 mg l^−1^), medium (21 mg l^−1^) or low (10.5 mg l^−1^) dose of (A) pyriproxyfen and (B) spirodiclofen that survived over a 768 h or 672 h period, respectively, with 95% confidence bands. Control bees were exposed to solvent only. For each treatment/dose combination, three replicate cages containing 50 bees each (*n*=150) were made from single-cohort bees that emerged on the same day. Comparisons between survival cages were conducted using log-rank tests. Dashed lines represent the median survival time (when 50% of bees perished) for the corresponding treatment.

Caged bees fed a high (42 mg l^−1^) concentration of pyriproxyfen at adult emergence exhibited higher mortality than those fed the negative (acetone only) control (χ^2^=27.2, *P*<0.0001; Fig. 2A), reaching 50% mortality at 408 h, or 17 days. Spirodiclofen-treated bees had no significant differences in median survival time between treatment groups or the negative control (χ^2^=4.5, *P*=0.22; Fig. 2B**)**.

### Foraging onset and behavior

**Fig. 3.**
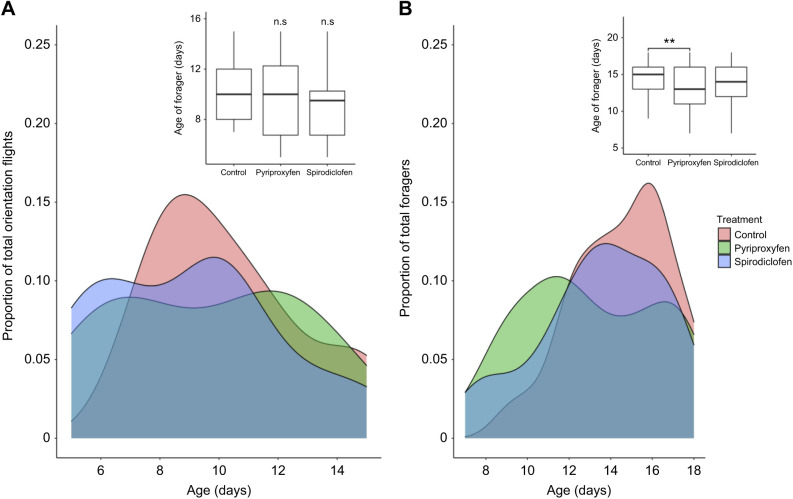
**Pyriproxyfen treatment lowers average age at first forage, but not onset of orientation flights.** Density plots indicating the frequency of observed (A) orientation flights (*n*=41) and (B) first foraging trips (*n*=190) for bees in each group on each day following pesticide treatment at adult emergence. Data were collected from 5 replicate hives. Inset: boxplots represent the mean age in days (middle line of boxplots) when orientation flights or first foraging trips occurred by group, with error bars representing the s.e.m. and box boundaries representing the interquartile range. Asterisks represent significant differences as determined by pairwise comparisons using Tukey's HSD *post hoc* test (**P<0.001; n.s., not significant).

Pesticide treatment did not influence the onset of orientation flights *(F*_2,38_=0.739, *P*=0.484; Fig. 3A). Forty-one orientation flights were observed, occurring as early as 5 days and as late as 15 days post-treatment. Pesticide treatment decreased the average age that bees were observed leaving the nest to forage (*F*_2,187_=4.66, *P*=0.0106; Fig. 3B). Pyriproxyfen-treated bees foraged significantly earlier than the control bees (Tukey's test: *t*_187_=−2.97, *P*=0.0096; Fig. 3B). There was no difference between the control and spirodiclofen groups in the age at first forage (Tukey's *t*-test: *t*_187_=−1.92, *P*=0.135). Although pyriproxyfen-treated bees foraged significantly earlier than the control, there were no significant differences between any groups in the ages at which foragers first returned with pollen (*F*_2,59_=0.727, *P*=0.488). We also found no significant effect of treatment (*F*_2,108_=0.210, *P*=0.811) or age (*F*_2,107_=0.495, *P*=0.482) on the likelihood that a forager returned with pollen versus water/nectar (Table 1). Because we found no significant differences in the prevalence of pollen foragers between treatments, we concluded that pesticide treatment was unlikely to influence the type of dietary resource (pollen versus water/nectar) collected by foragers.

**Table 1.**
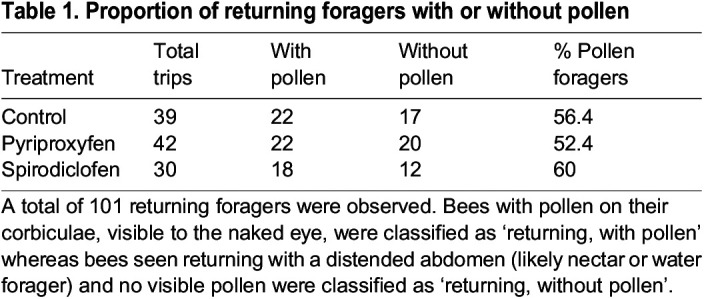
Proportion of returning foragers with or without pollen

### Forager abdominal and corbicular pollen lipid

**Fig. 4.**
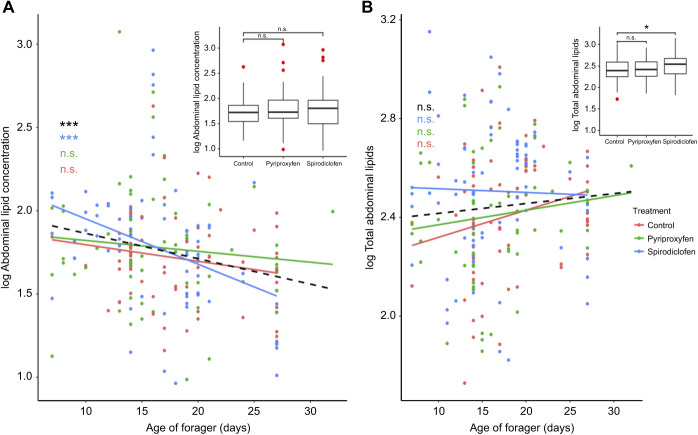
**Abdominal total lipids are greater, but decline significantly faster, following spirodiclofen treatment.** Scatter plots of (A) abdominal lipid concentration (μg total lipid mg^−1^ bee total tissue) and (B) total abdominal lipids (μg) by age of collected forager. Each data point represents a single collected forager's age and their corresponding lipid value. Colored lines (see legend) indicate stratified linear regression by treatment; black dashed lines indicate overall linear regression across all treatments. Significance of the linear regressions is indicated (***P<0.0001). Inset: boxplots represent the mean (middle line of boxplot) abdominal lipid concentration (A) and total abdominal lipid (B) by treatment, with error bars representing s.e.m., box boundaries representing the interquartile range, and red circles indicating outliers. Asterisks represent differences as determined by pairwise comparisons using Tukey's HSD *post hoc* test (*P<0.01).

We collected pollen foragers to examine the relationship between internal lipid levels and collected dietary lipid. There was a significant effect of pesticide treatment on total abdominal lipids (total μg) (*F*_2,203_=3.52, *P*=0.032), with spirodiclofen-treated bees initially having significantly higher average total abdominal lipid in comparison to controls (Tukey's test: *t*_203_=−2.479, *P*=0.037; Fig. 4B). Although treatment did not affect average abdominal lipid concentration (total μg mg^−1^ dry carcass mass; *F*_2,203_=0.736, *P*=0.480), we noticed a strong effect of day on abdominal lipid concentration (μg mg^−1^; *F*_2,203_=12.08, *P=*0.0006; Fig. 4A). When stratifying the model by treatment, we found that spirodiclofen-treated bees experienced a significantly accelerated decline in abdominal lipid concentration (*R*^2^=0.136, *F*_1,75_=12.98, *P*<0.001; Fig. 4A), which was not found in those treated with pyriproxyfen (*R*^2^=−0.00359, *F*_1,65_=0.764, *P*=0.385) or the control group (*R*^2^=0.0167, *F*_1,63_=2.09, *P*=0.153).

**Fig. 5.**
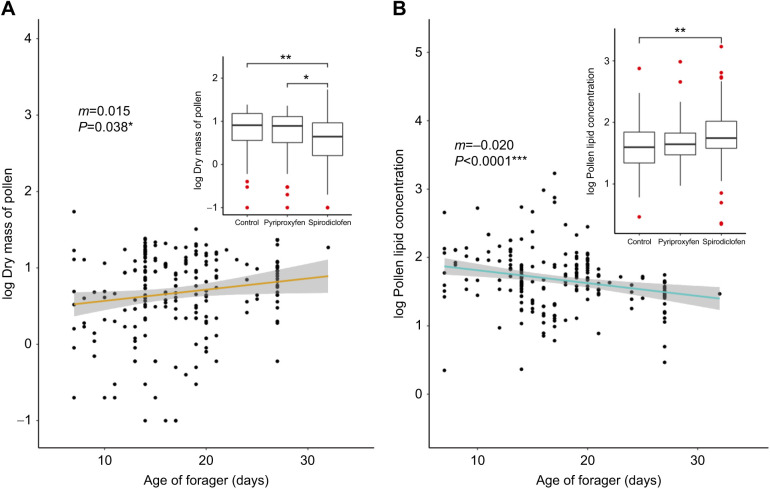
**Spirodiclofen treatment increases the amount and lipid concentration of collected pollen.** Scatterplots of (A) dry mass of collected pollen (mg pollen) and (B) pollen lipid concentration (μg pollen lipid mg^−1^ pollen) by age of collected forager. Each point represents corbicular pollen collected from a single forager and their age. Trendlines indicate the best-fit linear regression. Inset: boxplots represent the mean (middle line of boxplot) dry mass of pollen (A) and pollen lipid concentration (B) by treatment, with error bars representing s.e.m., box boundaries representing the interquartile range, and red circles indicating outliers. Asterisks represent differences as determined by pairwise comparisons using Tukey's HSD *post hoc* test (**P<0.001, *P<0.01).

**Fig. 6.**
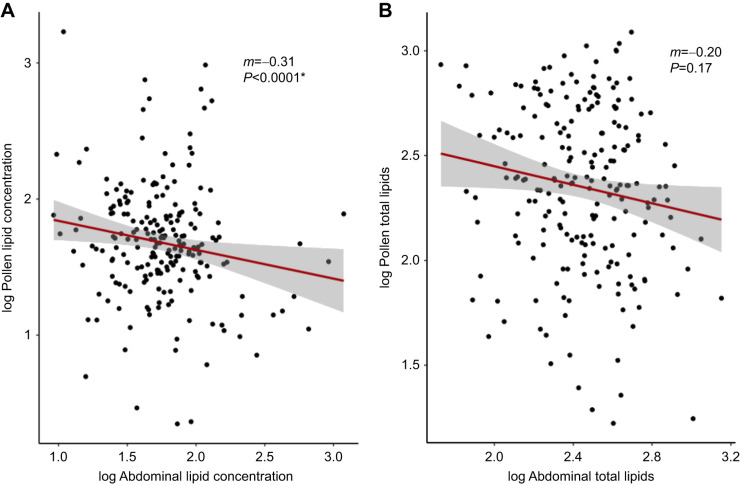
**Forager abdominal lipid concentration significantly declines with reduction in pollen lipid concentration.** Scatterplots of (A) pollen lipid concentration (μg lipid mg^−1^ pollen) by abdominal lipid concentration (μg total lipid mg^−1^ bee total tissue) and (B) total pollen lipid (μg) by total abdominal lipid (μg). Each point represents a single forager's (*n*=209) internal lipid state corresponding to the lipid quantity of their collected pollen. Red trendlines indicate best-fit linear regression with gray shading representing standard error.

Pesticide treatment influenced both the amount of pollen that a forager collected and the lipids in the pollen. Each forager returned with a mean (±s.e.m.) of 8.11±7.27 mg dry mass of pollen, with a lipid concentration of 85.6±161.9 μg lipid mg^−1^. Treatment influenced both the amount of pollen collected (mg; *F*_2,203_=4.660, *P=*0.0105) and the pollen's lipid concentration (μg mg^−1^; *F*_2,203_=3.05, *P*=0.0496) (Fig. 5). Spirodiclofen-treated bees returned with significantly less pollen compared with the controls (*P*=0.0122). However, the pollen that the spirodiclofen-treated bees collected had a higher lipid concentration (*P*=0.0383) compared with that collected by controls. Forager age did not affect the amount of pollen that each forager collected (mg; *F*_2,203_=3.46, *P=*0.0643), but it did affect its lipid concentration (μg mg^−1^; *F*_2,203_=11.7, *P*<0.0001). Older bees returned with pollen that was less concentrated in terms of lipids compared with younger bees. Foragers with less concentrated abdominal lipid collected less lipid-concentrated pollen (*R*^2^=0.110, *F*_6,202_=5.30, *P*<0.0001; Fig. 6A). Total abdominal lipid, however, was not an accurate predictor of total pollen lipid (*R*^2^=0.015, *F*_6,202_=1.52, *P*=0.171; Fig. 6B), suggesting that abdominal lipid concentration is a more accurate predictor of pollen dietary lipid.

## DISCUSSION

Our results provide a new mechanistic basis for the connection between stress, lipid homeostasis and behavior in honey bees. A single, acute stress exposure early in adulthood altered behaviors much later in life, as bees fed pesticides once as newly emerged workers exhibited behavioral changes later as foragers (roughly ≥10 days after treatment). The continuation of foraging for higher-value food items, even after the cessation of the stress response, is likely a conserved evolutionary response observed not only in vertebrates but also in invertebrate models. If this response is truly conserved, this insect system might be used to further study the links between stress and pathologies related to altered lipid homeostasis in humans.

* *Bees treated with the acute stressor pyriproxyfen, a juvenile hormone mimic, before they were then placed into a colony had a lower average age at first forage. This is consistent with existing literature on another juvenile hormone analog, methoprene, which also decreased the age at first foraging flight, as well as the effective age at death (Chang et al., 2015). Contrary to our predictions, precocious foraging in pyriproxyfen-treated individuals was not associated with expedited lipid depletion. For this reason, foraging onset is more likely a synergy of many hormonal and environmental factors, including (but not limited to) endogenous juvenile hormone titers (Sullivan et al., 2000). Because pyriproxyfen-treated bees were more likely to forage precociously, and precocious foragers are speculated to have sub-optimal foraging performance (Woyciechowski and Moroń, 2009; Ushitani et al., 2016), we expected them to collect less pollen (smaller corbicular pellets) per foraging trip. This was not the case: pyriproxyfen-treated foragers collected the same amount of pollen per trip as the control foragers. Lipid levels in the collected pollen were also equivalent between pyriproxyfen-treated bees and controls. From this study, we also cannot say with certainty whether pyriproxyfen-induced precocious foraging affects colony demography more broadly. Our initial assays of survival suggest that pyriproxyfen exacts some cost. If pyriproxyfen elevates forager mortality above a threshold level at the hive level, colony collapse remains a likely possibility (Perry et al., 2015; Khoury et al., 2011).

The results from the spirodiclofen-treated bees support a link between stress, lipid homeostasis and behavior. Previous literature used total abdominal lipid to measure changes in fat body lipid integrity (Toth et al., 2005; Anand and Lorenz, 2008). We also wanted to see whether lipid levels were affected as a proportion of individual bee weight, so we also analyzed the effect of pesticides on the abdominal lipid concentration (proportion of total bee biomass due to lipid). Spirodiclofen-treated bees had significantly higher total abdominal lipids compared with controls, but treatment did not increase abdominal lipid concentration. This suggests that, while spirodiclofen treatment may have a significant effect on increasing absolute lipid levels, it does not increase lipid levels as a proportion of total biomass. In fact, when we incorporated biomass into our model, we found that spirodiclofen-treated bees exhibited a significantly accelerated decline in abdominal lipid concentration not seen in other treatment groups. Spirodiclofen impedes lipid biosynthesis, so this accelerated depletion of abdominal lipid may be due to bees being incapable of replenishing their own lipid reserves. It is also possible that fatty acid synthesis inhibitors make younger bees hungrier because they deposit fewer fatty acids in their fat body, and so they overconsume stored nutrients in the hive to compensate. Later, as foragers, when they are less prone to overconsumption, they may utilize their lipid stores even faster, as seen in our forager-aged bees. This may explain the behavioral change of spirodiclofen-treated bees collecting less, yet fattier pollen. It is possible that stressors with a similar mode of action to spirodiclofen motivates bees to increase dietary lipid intake to maintain energetic homeostasis. We looked only at pollen foragers because pollen is the singular source of dietary lipid for honeybees and the resource most relevant to our overall question:– is there a relationship between internal lipid and collected dietary lipid? While this discounts the nectar and water foragers, the selection bias was found to be equal among treatment groups, as the percentage of pollen foragers (among all foragers) was equal across treatments (Table 1). Therefore, it was justifiable to subset our forager collection to only include pollen foragers. Within the pollen forager demography, we found that expedited lipid depletion leads to changes in pollen foraging behavior.

Ultimately, these results support our hypothesis that bees regulate lipid intake in response to stress. Fueling the stress response can be energetically costly (Arrese and Soulages, 2010), so it is reasonable that stressed colonies would collect fattier pollen to restore lipid homeostasis. Honey bees select pollen with macronutrient ratios that balance nutritional deficiencies (Corby-Harris et al., 2021; Hendriksma and Shafir, 2016) and optimize colony health and survival (Bouchebti et al., 2022). With this in mind, high-lipid diets may provide a benefit to bees challenged with pesticides or parasites (Annoscia et al., 2017; Crone and Grozinger, 2021). Given our current need for crop pollination, especially in agricultural landscapes where floral diversity is low and the risk of pesticide or parasite exposure is high (Goodrich et al., 2019), colonies might benefit from additional lipid-rich forage and/or supplemental diets during key periods before and after bloom.

The nutritional dearth experienced by bee colonies in agricultural landscapes with low floral diversity is analogous to the food scarcity that is experienced by people residing in communities with limited access to affordable, high-quality foods. These communities are typically more vulnerable to a plethora of stressful circumstances, including higher rates of poverty and violence (Walker et al., 2010). These communities also face a higher prevalence of obesity and other metabolic diseases (Ghosh-Dastidar et al., 2014). Our study reinforces the hypothesis that stress has lasting consequences on lipid homeostasis and food intake behavior. From a public health perspective, stress management should be an important factor when considering preventative measures against human diseases associated with altered lipid balance, such as obesity. The present study suggests that honey bees provide an experimental framework for addressing such questions on the stress–lipid–behavior axis.
